# Marsupialisation of a tunnel flap for a false oesophageal lumen post peroral endoscopic myotomy

**DOI:** 10.1055/a-2772-0195

**Published:** 2026-01-15

**Authors:** Hasib Ahmadzai, Clarence Kerrison, Jun Young Kim, Brian Lam, Yong Sul Kim, Sunil Gupta, Michael J. Bourke

**Affiliations:** 1 570073 Department of Gastroenterology and Hepatology, Westmead Hospital, Westmead, Australia; 2 216997 University of Sydney, Westmead Clinical School, Sydney, Australia

**Fig. 1:**
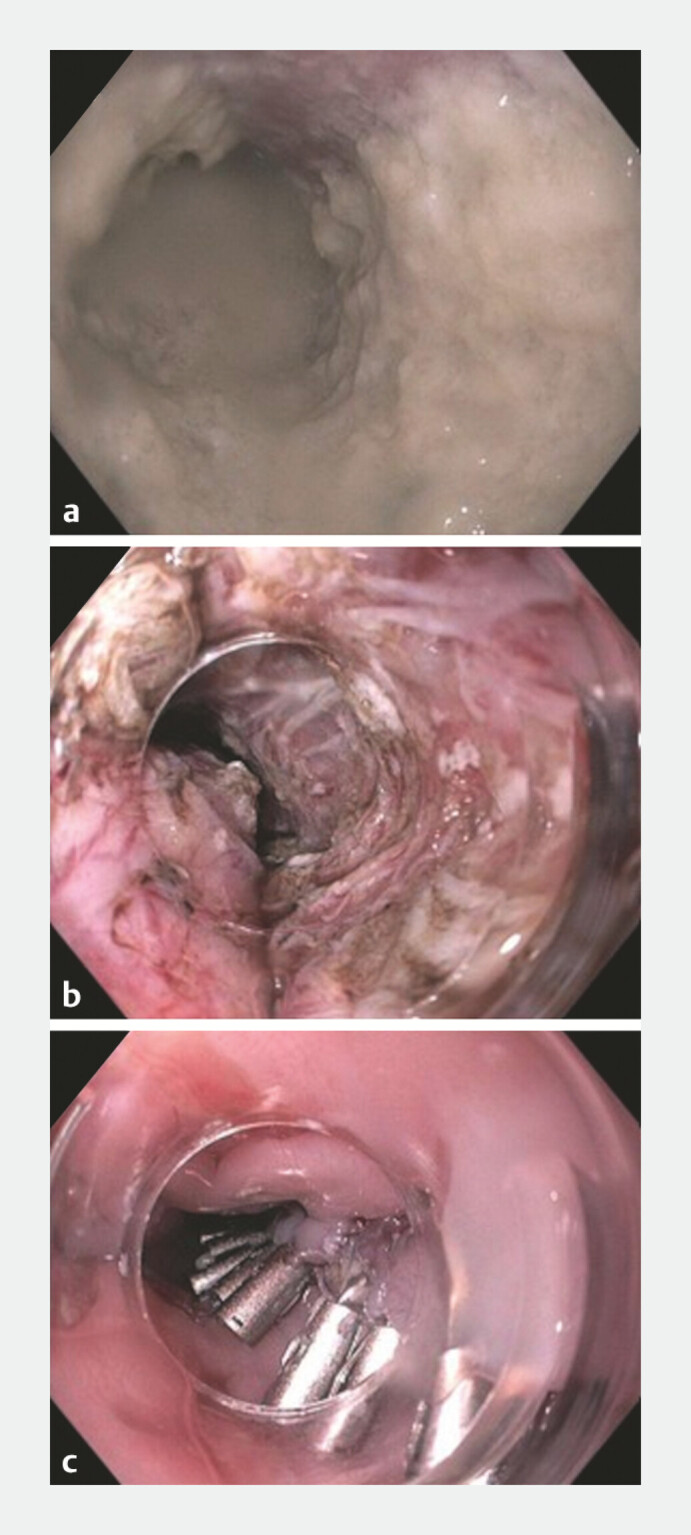
Achalasia changes with food contamination and aperistalsis in **a** . Peroral endoscopic myotomy performed in **b** , with clip closure following the POEM procedure in **c** . POEM, peroral endoscopic myotomy.

A 68-year-old woman with type 2 achalasia, chronic oesophageal stasis, a thickened mucosal layer, and an Eckardt score of 8 underwent an uncomplicated peroral endoscopic myotomy (POEM; Fig. 1 ). One-month post-POEM, she developed recurrent dysphagia, regurgitation and aspiration. Repeat gastroscopy revealed dehiscence of the oesophageal mucosotomy (tunnel orifice) and formation of a long false lumen ( Video 1 ). This was consistent with the original submucosal tunnel, with its wall being a mucosal flap and a healed post-myotomy muscularis propria layer.

Mucosotomy dehiscence is found 1 month following a POEM procedure. Marsupialisation of the tunnel mucosal flap is performed with resolution of the patient’s symptoms. POEM, peroral endoscopic myotomy.Video 1

**Fig. 2:**
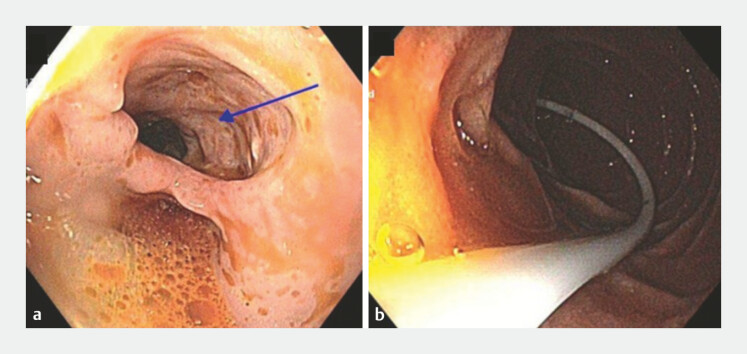
Formation of a false lumen from the open POEM tract (blue arrow) in **a** and insertion of a nasojejunal feeding tube in **b** . POEM, peroral endoscopic myotomy.

**Fig. 3:**
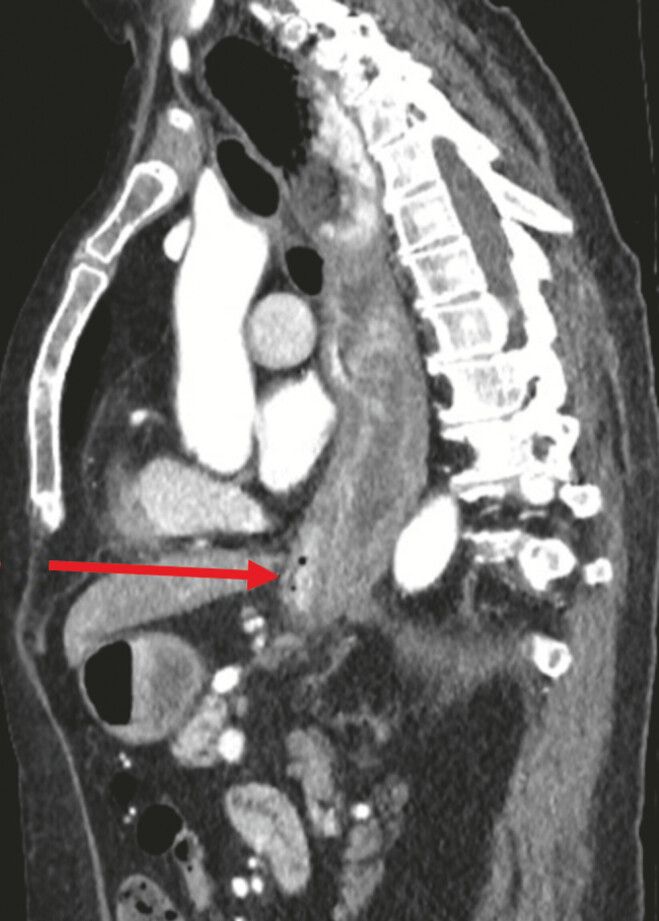
A sagittal CT scan with oral contrast showing no mediastinal leak with a dilated oesophagus (red arrow). CT, computed tomography.

**Fig. 4:**
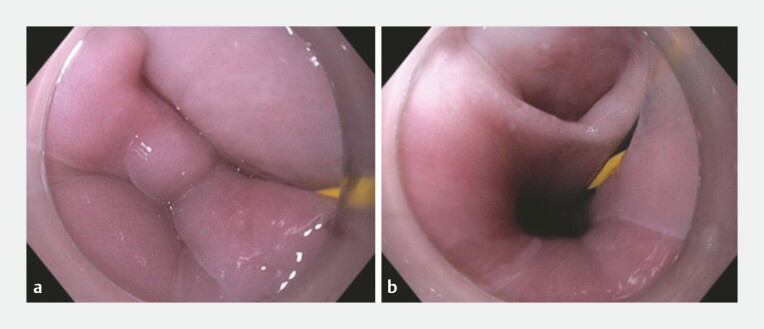
A false lumen with a Jagwire passing into the open POEM tract in **a** and insertion of the Jagwire through the POEM tract into the stomach in **b** . POEM, peroral endoscopic myotomy.

A nasojejunal feeding tube was endoscopically placed for feeding ( Fig. 2 ). A computed tomographic scan with oral contrast showed minimal passage of contrast into the distal third of the oesophagus and no mediastinal leak ( Fig. 3 ). Despite a conservative approach, repeat gastroscopy after a further 4 weeks demonstrated a persistent false lumen, a dilated proximal oesophagus containing food debris and oesophageal candidiasis ( Fig. 4 ).

**Fig. 5:**
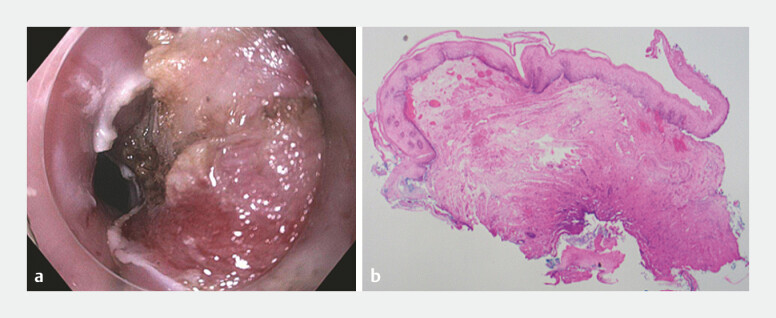
Marsupialisation of the tunnel flap in **a** and histology of the resection mucosal flap in **b** , showing dense fibrosis in submucosa with no definite muscularis propria seen.

Three months post-POEM procedure, we proceeded with endoscopic marsupialisation of the false lumen. The tunnel was completely epithelialised. There was a small fistula back into the oesophagus at the level of the gastro-oesophageal junction (GOJ). A 0.035-inch Jagwire was placed into the false lumen, through the distal opening and into the stomach ( Fig. 5 ). An endoscopic knife was used to dissect the mucosal flap, using the wire as a guide down to the GOJ. Redundant mucosal tissue at the site of the incision was then resected with the EMR technique using a 10 mm hot snare. Repeat gastroscopy 7 months post-POEM showed a healthy appearing scar without evidence of a false lumen. The lower oesophageal sphincter opened easily ( Video 1 ). Reassuringly, the patient’s symptoms had resolved following this, with an Eckardt score of 0.

Dehiscence of a submucosal tunnel mucosotomy site leading to a false lumen is a rare complication of POEM 1 2 3 . Symptom recurrence post-POEM warrants further endoscopic evaluation. Herein, we have demonstrated a novel technique of guidewire-assisted marsupialisation and eliminated the false lumen and the associated symptoms.

Endoscopy_UCTN_Code_TTT_1AQ_2AD_3AZ
